# Macronutrient Composition of the Diet Affects the Feeding-Mediated Down Regulation of Autophagy in Muscle of Rainbow Trout (O. *mykiss*)

**DOI:** 10.1371/journal.pone.0074308

**Published:** 2013-09-12

**Authors:** Ikram Belghit, Stéphane Panserat, Bastien Sadoul, Karine Dias, Sandrine Skiba-Cassy, Iban Seiliez

**Affiliations:** INRA, UR1067 Nutrition Métabolisme Aquaculture, St-Pée-sur-Nivelle, France; Van Andel Institute, United States of America

## Abstract

Autophagy functions as an important catabolic mechanism by mediating the turnover of intracellular organelles and protein complexes through a lysosome dependent degradative pathway. Although the induction of autophagy by starvation has been extensively studied, we still know very little about how autophagy is regulated under normal nutritional conditions. The purpose of the present study was to characterize both in vivo and in vitro the response of the autophagy-lysosomal degradative pathway to nutrient (amino acids and carbohydrates) availability in the muscle of the carnivorous rainbow trout. We report that meal feeding is accompanied by a rapid activation of Akt, FoxO1 and the Target of Rapamycin (TOR) signaling pathways and a concomitant decrease of autophagosome formation. We also show that this effect occurs only when the proportion of dietary proteins increases at the expense of carbohydrates. Concurrently, our in vitro study on primary culture of trout muscle cells demonstrates an opposite effect of amino acids and glucose on the regulation of autophagy-lysosomal pathways. More specifically, the addition of amino acids in cell culture medium inhibited the formation of autophagosomes, whereas the addition of glucose had an opposite effect. The effect of amino acids was accompanied by an activation of TOR, considered as an important regulator of autophagosomal formation. However, the mechanisms involved in the effect of glucose were independent of Akt, TOR and AMPK and remain to be determined. Together, these results demonstrated the specific role of macronutrients as well as that of their interactions in the regulation of autophagy and highlight the interest to consider the macronutrient composition of the diets in the control of this degradative pathway.

## Introduction

Protein degradation is a highly regulated and selective process that depends on the activation of conserved proteolytic pathways [1]. Two major proteolytic systems are thought to (co-) operate in the skeletal muscle of vertebrates, the ubiquitin/proteasomal pathway and the autophagic/lysosomal pathway [2]. The ubiquitin/proteasomal pathway has been well documented and has long been considered to be the primary system involved in muscle atrophy [3]–[7]. In contrast, until recently, the role of autophagy in skeletal muscle protein degradation has been largely ignored. Recent work by Masiero *et al.*, however, has demonstrated that basal autophagy is critical to muscle homeostasis, since it is responsible for the removal of protein aggregates and damaged mitochondria [8]. Accordingly, mice with muscle-specific suppression of autophagy exhibited severe muscle weakness, atrophy and decreased muscle contractility [8].

The autophagic/lysosmal pathway is a highly conserved homeostatic process that is responsible for the degradation of cytoplasmic components and recycling of long-lived proteins. During autophagy, a part of the cytoplasm is sequestered by a double membrane, called autophagosome. After its formation, the autophagosome undergoes fusion events with the lysosome and acquires hydrolytic enzymes favoring the rapid degradation of the sequestered material [9], [10]. One of the most widely monitored autophagy-related proteins is LC3 (Atg8 in yeast). In mammalian cells, cytosolic LC3 is synthesized as a precursor (Pro-LC3-I). Immediately after its synthesis, a C-terminal fragment is cleaved by Atg4 to produce LC3-I with an exposed glycine residue that binds covalently to phosphatidylethanolamine (PE) on the autophagosomal membrane to form LC3-II. Once the autophagosome is formed, LC3-II localizes both at the cytosolic and luminal faces of its double membrane. After fusion of the autophagosome with lysosome (autolysosome), the luminal LC3-II is degraded by lysosomal cathepsins, while Atg4 recycles LC3-I and PE from LC3-II on the cytosolic side of the autolysosome membrane. As such, LC3-II is the only protein marker that is reliably associated with the different steps of the autophagic process [11].

Regulation of autophagy has been extensively studied, but there are still many unknowns [10]. Under basal conditions, autophagy occurs at low levels in almost all cells, but it is strongly induced under stress conditions such as starvation, oxidative stress or infectious diseases [12]–[14]. Much attention has been paid to the mechanisms involved in the induction of autophagy during nutrient starvation [13], [15], [16]. In this regard, depletion of total amino acids has been shown to strongly induce autophagosome formation via the inactivation of the major nutrient sensing mammalian target of rapamycin (mTOR) protein [17]. However, in skeletal muscle, the importance of mTOR in autophagy regulation has been questioned [2] and several evidences identified the Akt-FoxO signaling axis as a more critical factor for autophagy control in this tissue [2], [18], [19]. Similarly, an important role has been reported for the energy sensing AMP activated protein kinase (AMPK) in autophagy induction in response to various cellular stresses, including glucose starvation [17], [20]–[23].

Less is known about how basal autophagy is regulated under normal nutritional conditions when nutrients are not limiting. However, this question is of particular importance given the role of basal autophagy as an important intracellular quality-control system [13]. To address this question and to gain insight into the role of nutrients and their interactions in the regulation of the basal autophagy, we analyzed the post-prandial response of the autophagosomal marker LC3-II as well as that of the upstream factors Akt, FoxO, TOR and AMPK following a single meal of different ratio of dietary carbohydrates/proteins in the muscle of the carnivorous rainbow trout (*Oncorhynchus mykiss*). Furthermore, in order to analyze more specifically the effects of amino acids and/or carbohydrates on the regulation of autophagy, in vitro studies were performed using primary cultures of trout muscle cells. Rainbow trout emerged as a relevant model organism in the investigation of the nutritional regulation of autophagy [11], [24], [25]. The unusual features of its nutritional metabolism compared to mammals (i.e., a high dietary protein requirement combined with an apparent inability to metabolize dietary carbohydrates), make this species also particularly pertinent in comparative physiology.

## Materials and Methods

### Ethics Statement

The experiments were carried out in accordance with the clear boundaries of EU legal frameworks, specifically those relating to the protection of animals used for scientific purposes (i.e. Directive 2010/63/EU), and under the French legislation governing the ethical treatment of animals (Decret no. 2001-464, May 29th, 2001). The investigators carrying out the experiment had “level 1” or “level 2” certification, bestowed by the Direction Départementale des Services Vétérinaires (French vetinary services) to carry out animal experiments (INRA 2002–36, April 14th, 2002). The experiment was conducted at INRA St Pée-sur-Nivelle, certified for animal services under the permit number A64.495.1 by the French vetinary services, which is the competent authority.

### Fish and Experimental Procedures

Prior to the experiments, fish (juvenile immature rainbow trout) were maintained in our own experimental facilities (INRA, Donzacq, France) in tanks kept in open circuits at 18°C with well-aerated water under natural photoperiod conditions and fed to visual satiety with a commercial diet (Skretting, France; crude protein: 49.8% dry matter, crude fat: 13.8% dry matter; gross energy: 22 kJ/g dry matter). Immediately prior to the experiments, fish were fasted for 48 h, in order to obtain the basal levels of plasma metabolites that are reached later in fish than in mammals due to slower intestinal transit and longer gastric emptying time at low temperatures compared with endothermic animals. Following the fast, fish were fed once *ad libitum* with the commercial diet or one of the three semi-purified diets of high (H), medium (M) or low (L) levels of protein (P) or carbohydrates (C) (HPLC, MPMC and LPHC, respectively) (Table 1). The amount of feed distributed per tank was measured to ensure that feed intake was similar between diets (around 2% of their body weight). The diets were marginal, adequate and in excess of rainbow trout protein requirements (18, 37 and 65%, respectively) (NRC, 2011). Six trout were sampled for each time point, starting with unfed fish at 0 h, and following feeding at 2 h, 4 h, 12 h and 24 h. Immediately following complete anesthesia, which was confirmed by a complete absence of breathing or swimming response, trout were killed by a sharp blow to the head and decapitated to ensure fish were dead. Gut content of the sampled animals was checked to verify that fish had effectively consumed the diet. Dorsoventral white muscle from each fish were dissected, weighed, and immediately frozen in liquid nitrogen and kept at −80°C.

**Table 1:** Composition of the experimental diets.

Ingredients (%)	HPLC	MPMC	LPHC
Casein^1^	0.572	0.310	0.154
Casein hydrolysat^2^	0.100	0.055	0.021
L-arginine^3^	0.013	0.007	0.004
Dextrine^4^	0.050	0.350	0.537
Soy Lecithine^5^	0.020	0.020	0.020
Fish Oil^6^	0.130	0.130	0.130
CaHPO4.2H20 (18%P)^7^	0.000	0.013	0.019
Attractant mix^8^	0.015	0.015	0.015
Min. premix^9^	0.050	0.050	0.050
Vit. Premix^10^	0.0500	0.050	0.050
***Analytical composition (%)***			
Dry Matter (DM) (%)	90.96	92.61	93.09
Proteins (%DM)	65.15	36.69	17.62
Lipids (%DM)	17.05	15.72	15.59
Energy (kJ/g DM)	24.16	21.75	20.58
NFE (Cbh)^11^	6.38	32.52	48.84

1 Casein (Sigma-Aldrich, USA).

2 Casein hydrolysat (Sigma-Aldrich, USA).

3 L-arginine (Sigma-Aldrich, USA).

4 Dextrine (Sigma-Aldrich, USA).

5 Soy Lecithine (Louis François, St Maur des Fossés, France).

6 Feedoil (North sea fish oil, Sopropèche, Boulogne-sur-Mer, France).

7 CaHPO4.2H20 (18%P) (Sigma-Aldrich, USA).

8 Glucosamine 0,5 g; Taurine 0,3 g; Betaine 0,3 g; Glycine 0,2 g; Alanine 0,2 g/100 g feed.

9 Mineral mixture (g or mg/kg diet) : calcium carbonate (40%Ca), 2.15 g; magnesium oxide (60% Mg), 1.24 g; ferric citrate, 0.2 g;potassium iodide (75% I), 0.4 mg; zinc sulphate (36% Zn), 0.4 g; copper sulphate (25% Cu), 0.3 g; manganese sulphate (33% Mib), 0.3 g; dibasic calcium phosphate (20%Ca, 18%P), 5 g; cobalt sulphate, 2 mg; sodium selenite (30% Se), 3 mg; KCl, 0.9 g; NaCl, 0.4 g (UPAE (unité de préparation des aliments expérimentaux, Jouy, Inra, France)).

10 Vitamin mixture (IU or mg/kg diet): DL-a tocopherol acetate, 60 IU; sodium menadione bisulphate, 5 mg; retinyl acetate, 15 000 IU; DL-cholecalciferol, 3000 IU; thiamin, 15 mg; riboflavin, 30 mg; pyridoxine, 15 mg; B12, 0.05 mg; nicotinic acid, 175 mg; folic acid, 500 mg; inositol, 1000 mg; biotin, 2.5 mg; calcium panthotenate, 50 mg; choline chloride, 2000 mg(UPAE (unité de préparation des aliments expérimentaux, Jouy, Inra, France)).

11 Nitrogen-free extract (Carbohydrate) : 100– (crude protein+crude fat+crude fiber+moisture+ash).

### Plasma Metabolites

Plasma triglyceride levels were determined using a commercial kit (Biomérieux, France) adapted to a 96-microplate reader. Total plasma free amino acid (FAA) levels were determined by the ninhydrin reaction according to Moore [26] with glycine as standard.

### Cell Cultures

Primary culture of muscle cells were prepared from rainbow trout maintained in our experimental farm (Donzacq, France). Myoblasts were carried out using, for each culture, 30 to 60 animals, each weighing approximately 5 g. Cells were isolated from the latero dorsal muscle, pooled, and cultured following a previously described protocol [27]. Briefly, after removal of the skin, dorsal white muscle was isolated under sterile conditions and collected in Dulbecco’s modified Eagle’s medium (DMEM) containing 9 mM NaHCO_3_, 20 mM HEPES, 15% horse serum, and antibiotic-antimycotic cocktail (100 U/ml penicillin, 100 µg/ml streptomycin, and 0.25 g/ml fungizone) at pH 7.4. After mechanical dissociation of the muscle in small pieces, the tissue was enzymatically digested with a 0.2% collagenase (Sigma, C-9891) solution in DMEM for 1 h at 18°C and gentle shaking. The suspension was centrifuged (300 *g* for 5 min at 15°C), and the resulting pellet was subjected to two rounds of enzymatic digestion with a 0.1% trypsin solution in DMEM for 20 min at 18°C with gentle agitation. After each round of trypsinization, the suspension was centrifuged, and the supernatant was diluted in two volumes of cold DMEM supplemented with 15% horse serum (Sigma, H1270) and the same antibiotic-antimycotic cocktail mentioned above. After two washes with DMEM, the cellular suspension was filtered through 100- and 40-µm nylon filters. All experiments were conducted with cells seeded at a density of (160.000/cm^2^), in 12-well plastic plates (Nunc, 140675), and left for 30 min before medium change. Plates and coverslips were previously treated with poly-L-lysine (Sigma, P6282) and laminin (Sigma, L2020) to facilitate satellite cell adhesion. Cells were incubated at 18°C, the optimal temperature for culture of trout origin, with a complete medium containing Earle’s Balanced Salt (EBSS) culture medium (Sigma, E7510) supplemented with 10% fetal bovine serum (Sigma, F7524), MEM vitamins solution (Invitrogen, 11120-037), MEM essential amino acid mixture (Invitrogen, 11130-036) and MEM non-essential amino acid mixture (Invitrogen, 11140-035) and antibiotic-antimycotic cocktail under an air atmosphere. The medium was renewed every 2 days, and observations of morphology were regularly made to control the state of the cells.

### Treatment Conditions

After 4 days of culture, the cells were incubated in a minimal medium (MM) (Earle’s balanced salt solution with MEM vitamins and 5 mM glucose) supplemented or not with two fold concentrated amino acids (MEM as reference) and/or 25 mM Glucose in presence or absence of 100 nM of Bafilomycine A1 (Sigma, B1793). In experiments involving rapamycin (a specific TOR-inhibitor), the inhibitor (Cell Signaling Technologies, 9904) was added 30 min prior stimulation with AA. After 30 min or 4 h of incubation, the medium was removed, the wells were washed with ice-cold PBS and the cells were used for western blot analysis. Each experiment was performed at least two times.

### Protein Extraction and Western Blotting

Protein homogenates from muscles and cells were prepared as previously described [27]. Protein concentrations were determined with the Bradford reagent method [28]. Muscle or cell lysates (40 µg and 10 µg of protein, respectively) were subjected to SDS-PAGE and western blotting using the appropriate antibody: Anti-phospho Akt (Ser473) (Cell Signaling Technologies, 9271), anti-Akt (Cell Signaling Technologies, 9272), anti-phospho-p70-S6 kinase (Thr 389) (Cell Signaling Technologies 9205), anti p70-S6 kinase (Cell Signaling technologies 9202), anti-phospho AMPK (Thr172) (Cell Signaling Technologies, 2531), anti-AMPK (Cell Signaling Technologies, 2532), anti–LC3B (cell signaling Technologies, 2775), Anti-β-actin (Santa-Cruz Biotechnology, sc-47778), anti-phospho FoxO1 (Thr24)/FoxO3 (Thr32) (Cell Signaling 9464), anti FoxO1 (Epitomics, 1874-1), anti-β-tubulin (cell Signaling Technologies, 2146). All primary antibodies used have been shown to cross-react successfully with rainbow trout proteins of interest [25], [27], [29]–[31]. After washing, membranes were incubated with an IRDye infrared secondary antibody (LI-COR Inc. Biotechnology, Lincoln, NE, USA). Bands were visualized by Infrared Fluorescence using the Odyssey® Imaging System (LI-COR Inc.) and quantified by Odyssey infrared imaging system software (Application software, version 1.2).

### Statistical Analysis

All data were tested for homogeneity of variances by Bartlett tests, and then submitted to a one-way ANOVA (or two way-ANOVA for the combined effect of Bafilomycine A1 with amino acid or/and glucose in trout muscle cell), using R version 2.14.0. When data did not meet the assumptions of ANOVA, the non-parametric ANOVA equivalent (Kruskal–Wallis test) was performed. When these tests showed significance, individual means were compared using Tukey multiple-range tests. Significant differences were considered when P<0.05.

## Results

### Refeeding Induces Akt, FoxO and TOR Signaling Proteins and Inhibits Autophagy in Muscle of Trout

**Figure 1:**
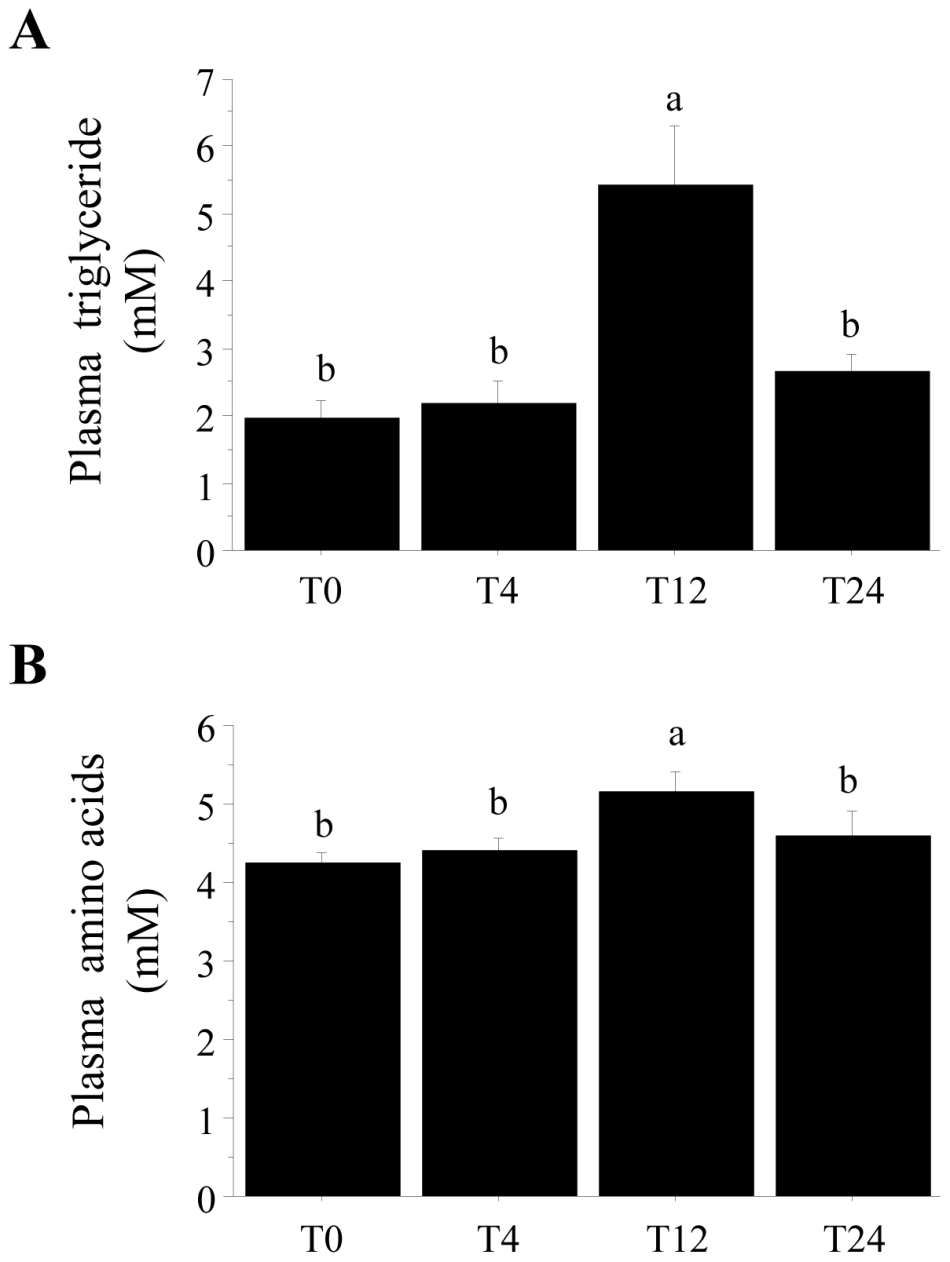
Plasma triglyceride (A) and amino acids (B) levels in rainbow trout fasted for 48 h (T0) and refed ad libitum with a commercial diet and sampled 4, 12 and 24 h after the meal. Results are means ± SE (n = 6). Different letters represent significantly different values (p<0.05).

**Figure 2:**
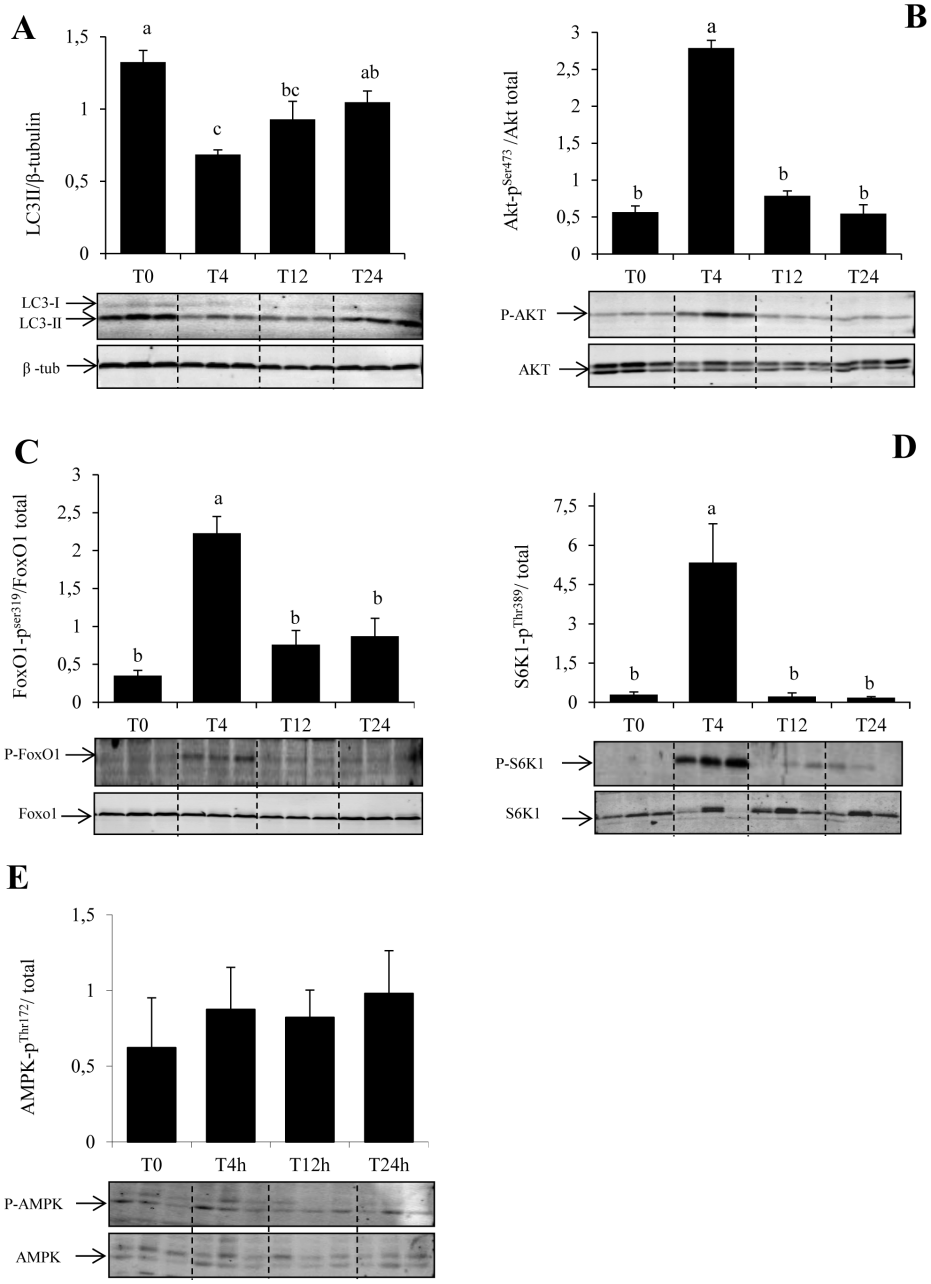
Postprandial response of the autophagosomal marker LC3-II and Akt, FoxO1, S6K1 and AMPK signaling proteins in trout muscle. Six trout were sampled for each time point, starting with unfed fish (T0) and following feeding at 4, 12 and 24 h. Muscle lysates (40 µg) were analysed by Western blot with the indicated antibodies. Representative blots are shown. For LC3b, the graph represents the ratio between LC3-II (lipidated form of LC3b) and β-tubulin used as loading control. For other proteins, graphs represent the ratio between the phosphorylated protein and the total amount of the targeted protein. Results are means ± SE (*n = *6). Different letters represent significantly different values (p<0.05).

Although the induction of autophagy by starvation has been extensively studied both in vivo and in vitro [15], [32], we still know very little about how basal autophagy is regulated under normal nutritional conditions. Here, we analyzed the postprandial response of the autophagosomal marker LC3-II as well as that of its upstream factors Akt, FoxO1, TOR and AMPK to a single meal in the muscle of rainbow trout. Validation of the “fasting-refeeding” experimental design was first performed by assessing the postprandial plasma triglyceride (TG) and free amino acids (AA) levels. As expected, refeeding induced a significant rise in plasma TG and AA levels 12 h after refeeding (Figs. 1A and B). Accordingly, the ratio LC3-II/β-tubulin fall by half as early as 4 h after the meal and remained significantly lower than that of unfed trout until 12 h after refeeding (Fig. 2A). Concomitantly, the phosphorylation of Akt, FoxO1 as well as that of the TOR effector S6K1 was significantly induced 4 h after the meal before returning to basal levels (Figs. 2B, C and D). In contrast, we did not find any effect of the nutritional status on the phosphorylation of AMPK (Fig. 2E). Together, these results show for the first time the postprandial response of autophagy to feeding in the muscle of rainbow trout.

### Macronutrient Composition of the Diet Affects the Feeding-mediated down Regulation of Autophagy in Muscle of Trout

**Figure 3:**
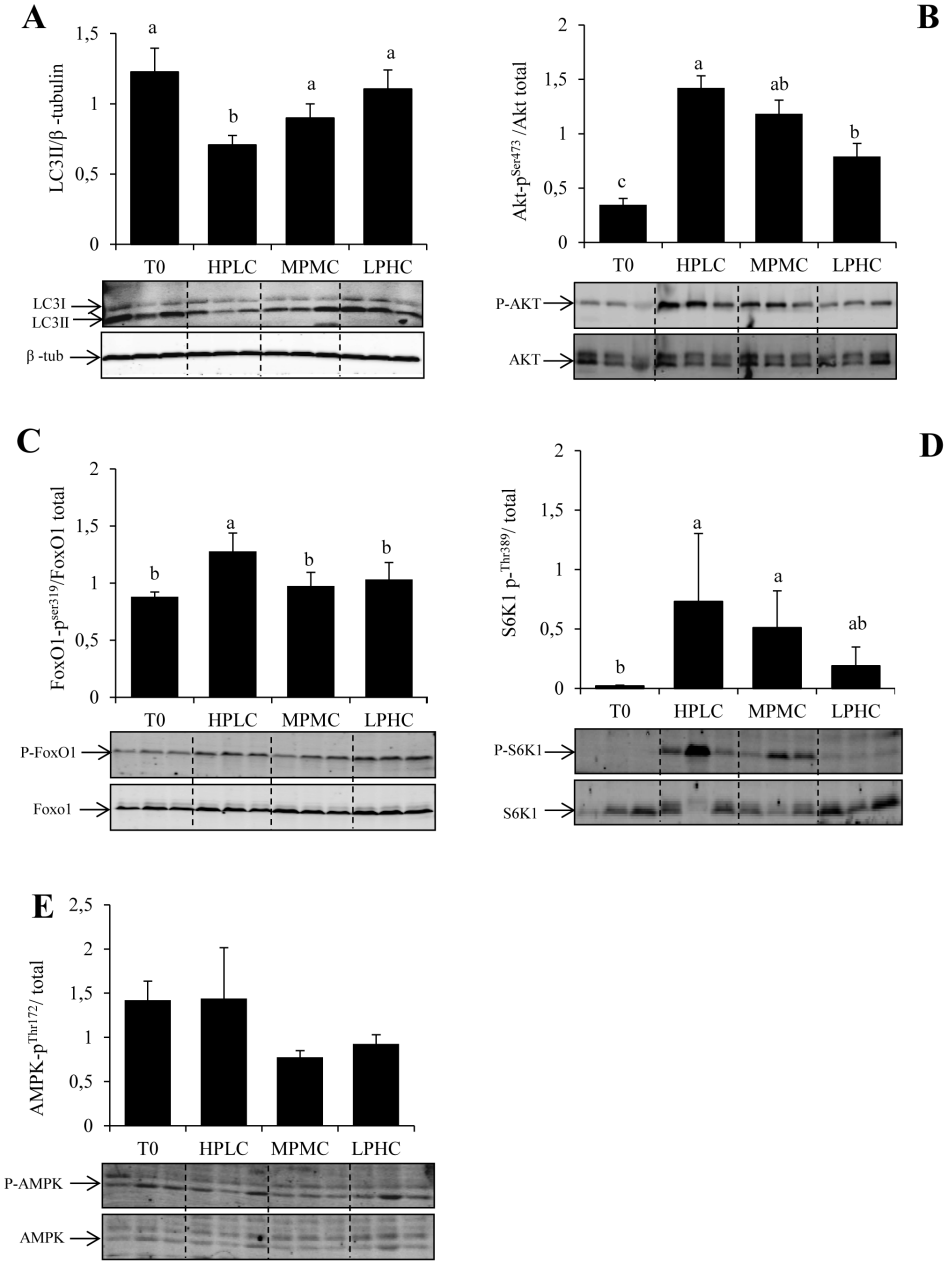
Western blot analysis of LC3-II processing and Akt, FoxO1, S6K1 and AMPK phosphorylation in fasted (T0) and HPLC, MPMC and LPHC diets 2h-refed rainbow trout. Muscle lysates (40 µg) were analysed by Western blot with the indicated antibodies. Representative blots are shown. For LC3b, the graph represents the ratio between LC3-II (lipidated form of LC3b) and β-tubulin used as loading control. For other proteins, graphs represent the ratio between the phosphorylated protein and the total amount of the targeted protein. Results are means ± SE (*n = *6). Different letters represent significantly different values (p<0.05).

We next addressed the question of the consequences of manipulating macronutrient composition of the diet in the regulation of muscle autophagy. More specifically, we investigated the effect of feeding three experimental diets of high (H), medium (M) or low (L) levels of protein (P) or carbohydrates (C) (HPLC, MPMC and LPHC, respectively) on the autophagosomal marker LC3-II as well as that of its upstream factors Akt, FoxO1, TOR and AMPK by comparing fasted and 2 h refed fish**.** As shown in Fig. 3A, the ratio LC3-II**/**β-tubulin reached significantly lower level in fish fed the HPLC diet compared with fasted trout. In contrast, no significant decrease was observed in fish fed the MPMC and LPHC diets. Likewise, the analysis of the phosphorylation of Akt, FoxO1 and S6K1 showed that HPLC was the only diet significantly affecting each protein (Figs. 3B, C and D). The phosphorylation of AMPK showed no significant differences among dietary groups (Fig. 3E). Overall, these data demonstrated for the first time that the macronutrient composition of the diet may affect the autophagic response to feeding and suggest an involvement of TOR and/or Akt-FoxO1 signaling axis in this effect.

### Antagonistic Role of Amino Acids and Glucose in the Regulation of Autophagy in Trout Muscle Cell Culture

**Figure 4:**
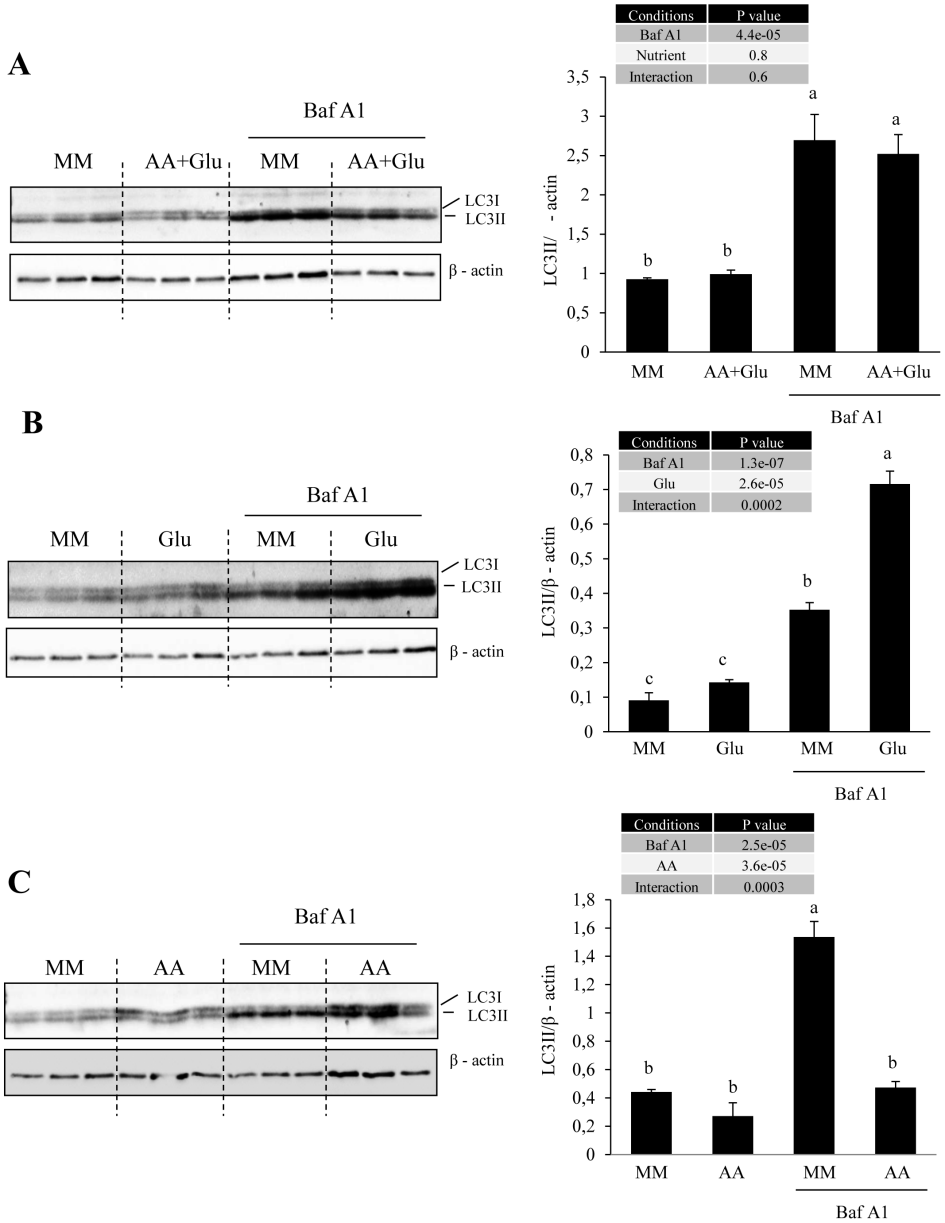
Antagonistic role of amino acids and glucose in the regulation of autophagy in trout muscle cell culture. Four-day-old cells were incubated in minimal medium (MM) supplemented or not with amino acids (AA) and/or 25 mM Glucose (Glu) in presence or absence of Bafilomycine A1 (Baf A1). After 4 h of incubation, cell lysates (10 µg) were analyzed by western blot with the indicate antibodies. The representative blot is shown. Graphs represent the ratio between LC3-II and β-actin. Results are means ± SE, *n = *3 (mean of 2 replications). Different letters represent significantly different values (p<0.05).

In order to clarify the specific and combined role of amino acids and glucose in the above presented feeding-induced repression of muscle autophagy, primary cultures of trout muscle cells were used. More specifically, we monitored by western blotting the LC3 lipidation in 4-day old cells stimulated or not with amino acids (AA) and/or glucose (Glu) in presence or absence of Bafilomycin A1 (Baf A1), a vacuolar ATPase inhibitor that inhibits autophagosome-lysosome fusion and prevents the degradation of LC3-II [11]. As expected, addition of Baf A1 increased the ratio LC3-II/β-actin (Figs. 4A, B and C), validating its use in our cell culture model. Addition of AA and Glu had no effect on the ratio LC3-II/β-actin, whatever the presence or the absence of BafA1 (Fig. 4A). When considering the effect of nutrients separately, we found a stimulatory effect of Glu on the ratio LC3-II/β-actin in presence of Baf A1 (Fig. 4B). In contrast, addition of AA in cells with Baf A1 decreased the ratio LC3-II/B-actin (Fig. 4C).

### Mechanisms Involved in AA- and Glu-mediated Regulation of Autophagy in Trout Muscle Cell Culture

**Figure 5:**
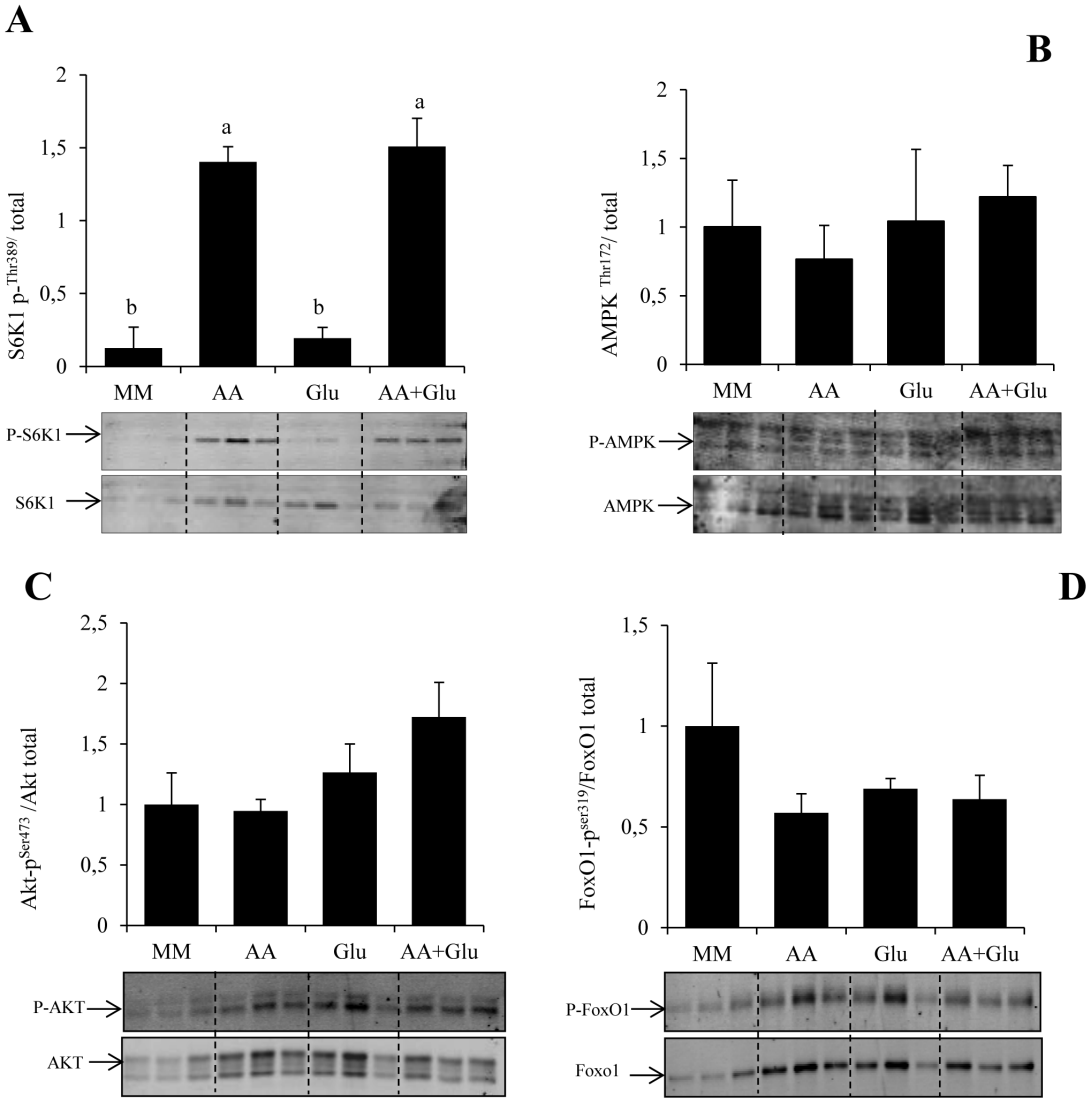
Mechanisms involved in AA- and Glu-mediated regulation of autophagy in trout muscle cell culture. Four-day-old cells were incubated in minimal medium (MM) supplemented or not with amino acids (AA) and/or 25 mM Glucose (Glu). After 30 min incubation, cell lysates (10 µg) were analyzed by western blot with the indicated antibodies. A representative blot is shown. Graphs represent the ratio between the phosphorylated protein and the total amount of the targeted protein. Results are means ± SE, n = 3 (mean of 2 replications). Different letters represent significantly different values (p<0.05).

In order to gain insight in the mechanisms involved in the above demonstrated effects of nutrients in LC3 lipidation, we monitored the phosphorylation of Akt, FoxO1, AMPK and the TOR effector S6K1 in cells stimulated or not with AA and/or Glu. The phosphorylation of S6K1 significantly increased in AA and AA+Glu treated cells but not in Glu treated cells (Fig. 5A). In contrast, no variation of Akt, FoxO1 and AMPK phosphorylation was monitored whatever the treatment (Figs. 5B, C and D). Altogether, these results suggest that, in our cell culture model, AA inhibit autophagy by activating TOR. However, the mechanisms involved in the effect of glucose seem to be independent of TOR, Akt-FoxO1 and AMPK and remain to be determined.

**Figure 6:**
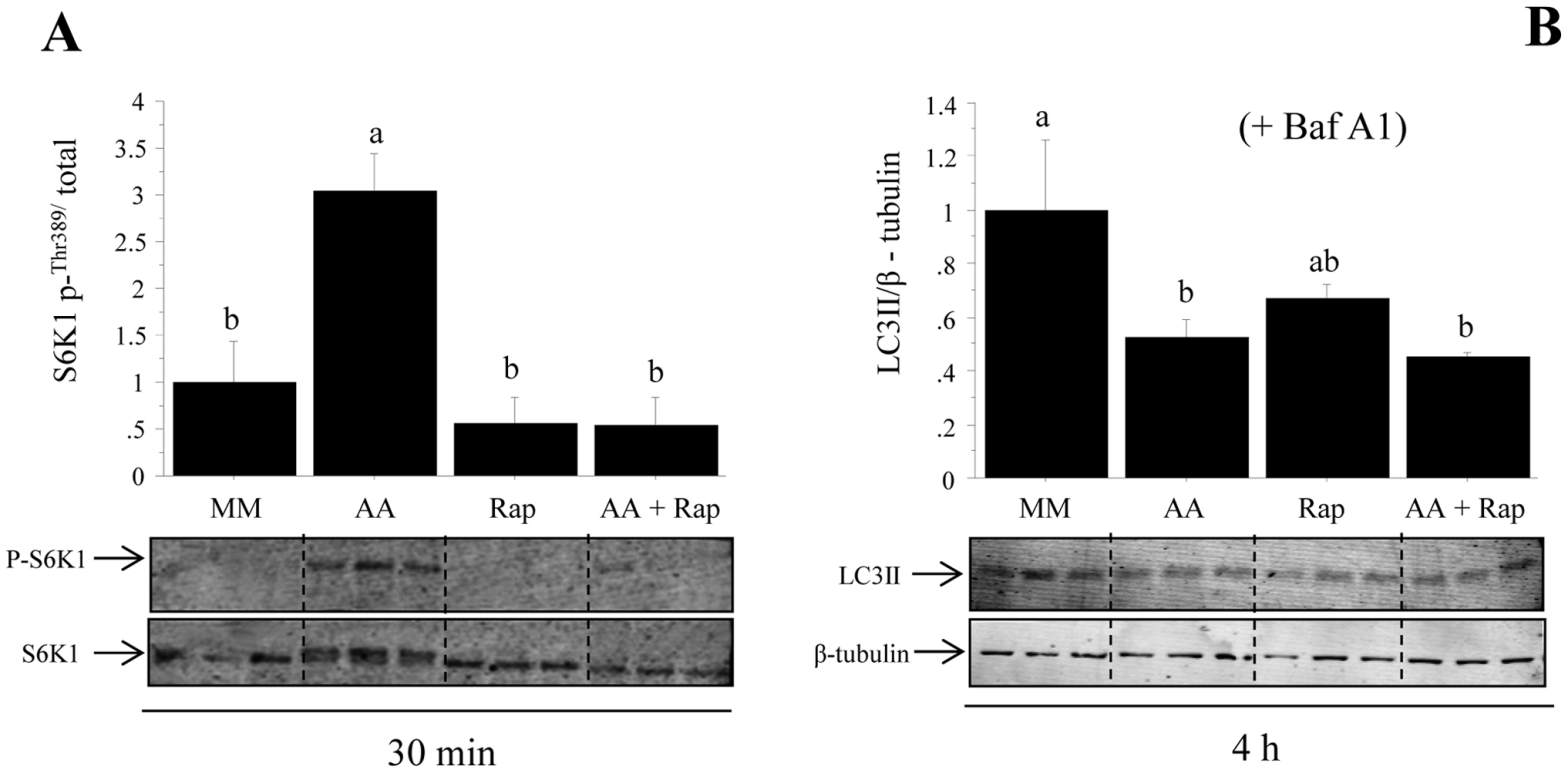
Effect of AA with or without rapamycin on S6K1 phosphorylation and LC3 lipidation in trout muscle cell culture. Four-day-old cells were preincubated for 30 min with or without 100 nM rapamycin. The culture mediums were then remplaced for 30 min or 4 h with the amino acid free medium (minimal medium group, MM and rapamycin group Rap) or a medium containing amino acids (amino acids group AA and amino acids plus rapamycin group AA-Rap) with or without Bafilomycine A1 (Baf A1). Cell lysates (10 µg) were analyzed by western blot with the indicated antibodies. Graphs represent the ratio between the phosphorylated protein and the total amount of S6K1 (A) and the ratio between LC3-II and β-tubulin (B). Results are means ± SE, n = 6 (mean of 2 replications). Different letters represent significantly different values (p<0.05).

In order to investigate if TOR mediates the above presented effect of AA addition on autotophagy, cells were then stimulated with AA in the presence or absence of the TOR inhibitor rapamycin for 30 min or 4 h. We first checked the specificity of rapamycin in our cell culture model by monitoring by western blot the phosphorylation of S6K1. As shown in Fig. 6A, the AA-mediated induction of phosphorylation of S6K1 was strongly abolished in the presence of rapamycin, demonstrating that rapamycin treatment specifically inhibits the activity of TOR in our cell culture model. We then analyzed the effect of AA with or without rapamycin on LC3 lipidation in presence of Baf A1 for 4 h. Our results showed that the inhibitory effect of AA on the ratio of LC3-II/β-tubulin was impaired in the presence of rapamycin to reach a non-significant value (Fig. 6B, compare lane Rap and AA+Rap). This indicated an involvement of the TOR protein in the effect of AA on autophagy. However, the surprising inhibitory effect of rapamycin alone on LC3-II (compare MM and Rap), although not significant, could weaken the observed effect of AA, making possible the involvement of other mechanisms in the observed effect of AA on autophagy.

## Discussion

Under physiological conditions, a basal autophagy is operating constantly for controlling the quality of proteins and organelles inside the cells [8], [33], [34]. This catabolic pathway can also be strongly induced under stress conditions, particularly in response to nutrient starvation [13], [35]. Most studies focused on the mechanisms involved in nutrient starvation induced autophagy, but less attention has been paid on autophagy regulation under normal nutritional conditions when nutrients are not limiting. Therefore, the aim of the present work was to characterize both in vivo and in vitro, the response of the autophagy/lysosomal pathway to the availability of dietary amino acids and carbohydrates in the muscle of the carnivorous rainbow trout.

We first analyzed the postprandial response of the autophagosomal marker LC3-II as well as that of its upstream factors Akt-FoxO, TOR and AMPK to a single meal in the muscle of rainbow trout. The obtained results show a decrease of the ratio LC3-II/β-tubulin as early as 4 h after the meal. To our knowledge, this is the first study showing the postprandial evolution of LC3B lipidation. The obtained results are somewhat surprising in comparison to the late (10–12 h) response of the Ubiquitin-Proteasome-dependent proteolytic system to refeeding as reported in trout [36] and in rats [37]. However, the activity of this last degradative system has been shown to be closely related to the “long term” transcription dependent control of the expression of several E3-Ubiquitine ligase coding genes [7], whereas that of autophagy has been reported to be also under a “short term” transcription independent control of signaling proteins, including TOR, AMPK [38]. In this regard, the results presented here show a concomitant increase of the phosphorylation of the TOR effector S6K1 4 h after meal feeding, suggesting that the TOR protein is activated at 4 h and making possible its involvement in the observed “short term” decrease of LC3-II levels. In contrast, we did not observe any effect of meal feeding on the phosphorylation of AMPK, excluding a possible involvement of this protein in LC3-lipidation.

We next addressed the question of the consequences of manipulating macronutrient composition of the diet in the regulation of muscle autophagy. More specifically, we investigated the effect of feeding three experimental diets of high, medium or low levels of protein or carbohydrates (HPLC, MPMC and LPHC, respectively) on LC3-II and its upstream factors Akt, FoxO1, TOR and AMPK. The obtained results demonstrated that macronutrient composition of the diet may affect the autophagosomal response to feeding. More specifically, we observed that LC3-II levels in the muscle of rainbow trout were not inhibited by refeeding when the proteins/carbohydrates ratio was severely decreased. These results are in close agreement with previous data in mice showing that feeding a protein deficient diet for several months activates autophagy [39]–[41]. However, here we demonstrate that, at least in rainbow trout, this effect occurs as early as the first day of feeding.

In order to clarify the specific and combined role of amino acids and glucose in the observed effect of macronutrient composition of the diet on LC3-II processing, primary cultures of trout muscle cells were incubated with or without amino acids and glucose. The obtained results showed that addition of amino acids in cell culture medium inhibited the formation of autophagosome. These results are in good agreement with previous evidences demonstrating that autophagosome formation is under the control of amino acid availability [35], [42]–[47], and suggest that the reduction of protein proportion in MPMC and LPHC diets likely contribute to attenuate the refeeding inhibition of LC3-II lipidation. The unexpected stimulatory effect of glucose on LC3-II processing in our in vitro trial is another point worthy of attention. Indeed, most studies demonstrated that glucose deprivation (and not addition) induces the autophagic/lysosomal pathway in mammalian cells [48], [49]. The only findings associating elevated glucose levels with induction of autophagy concerned the hyperglycemia-associated induction of autophagy in diabetic models [50], [51]. Interestingly, rainbow trout is considered to be glucose intolerant, and is characterized by a prolonged hyperglycemia following a glucose load or carbohydrate-rich meal [52]. The induction of LC3-II levels observed after glucose addition in cell culture media could therefore be a consequence of the specificity of the metabolism of the studied species. Furthermore, this effect could also contribute to the lack of MPMC- and LPHC-induced autophagosome formation arrest.

It has been proposed that the control of autophagy might involve TOR and AMPK signaling proteins [16], [17], [53]. In our experiment, we observed that the phosphorylation of the TOR effector S6K1 was increased when the levels of dietary proteins were augmented, a nutritional condition leading to a decrease of autophagy. In line with these results, our in vitro study demonstrated that treatment of trout myoblasts with the TOR inhibitor rapamycin impairs the AA mediated inhibition of autophagy. The inhibitory function of TOR in autophagy is well established for several tissues and cell culture systems [16], [54]. Genetic and pharmacological studies have shown that inhibition of TOR triggers activation of autophagy [55]. However, Sandri and co-workers demonstrated that the translation of these findings to skeletal muscle did not result in similar conclusion at least in mammals [2]. They demonstrated that TOR signaling is not the major regulator of autophagic flux in muscle and identified the Akt-FoxO pathway as a more critical signaling axis for autophagy control in this tissue [18], [19], [56]. In contrast, very recently Castets et al showed that sustained activation of TOR activity led to the development of a late-onset myopathy related to impaired autophagy, despite increased FoxO3 activity [57]. They concluded that TOR is the dominant regulator of autophagy in skeletal muscle. Our results show that rapamycin treatment of trout myoblasts prevents the AA mediated inhibition of autophagy, tipping the scales in favour of an important role of TOR in the regulation of autophagy in trout muscle.

Less is known about the mechanisms involved in the glucose-induced autophagy. In mammals, AMPK has been reported to play an important role in the regulation by glucose availability of autophagosome formation [17]. In the present study, we did not observe any significant change of the phosphorylation of AMPK in the tested conditions, excluding a possible involvement of this protein in the observed regulation of autophagy. However, the experimental conditions tested in the present study (5 mM glucose *vs* 25 mM glucose) are different to those showing the involvement of AMPK in the autophagic process that proceeded to a complete starvation of glucose [17]. Another hypothesis of the observed effect could be related to the oxidative stress generated by high glucose concentration [58]. Under these oxidative stress conditions, autophagy could constitute an adaptive pathway that promotes cell survival through the elimination of damaged proteins and organelles. In this regard, it has been shown in diabetic mammalian models that hyperglycemia-associated oxidative stress induces autophagy in several cell types [50], [59]. However, the mechanism involved in these effects remains largely unknown.

### Conclusion and Physiological Significances

In conclusion, the present results demonstrated that proteins/carbohydrates ratio of the diet affects the feeding-mediated down regulation of autophagy in muscle of rainbow trout. More specifically, we show that autophagosomal formation in the muscle of this species was not inhibited by refeeding when the proteins/carbohydrates ratio was severely decreased. One of the consequences of these results could be the observed prolonged hyperglycemia in trout fed high carbohydrates diets. Indeed, recent findings demonstrated that autophagy makes a significant contribution to the maintenance of blood glucose by converting amino acids to glucose via gluconeogenesis [60]. A lack of autophagy inhibition in trout fed high carbohydrates diet could therefore result in a prolonged release of gluconeogenic amino acids leading to the observed hyperglycemia. However, further studies are warranted to verify this hypothesis.

Overall, these results highlight the interest of the carnivorous rainbow trout as a model to gain better understanding of nutritional regulation of autophagy and the related consequences at physiological levels, especially in regard to its unusual metabolic features (i.e., a high dietary protein requirement, an important use of amino acids as energy sources, an apparent inability to metabolise dietary carbohydrates). From a practical aquaculture point of view, this study has additional importance as it will undoubtedly contribute to a better understanding of the nutritional regulation of a main degradative system in muscle and will provide a framework for future investigations in farmed fish species.
